# Supplementary material for: Incomplete inhibition of HIV infection results in more HIV infected lymph node cells by reducing cell death
Source: eLife. 2018 Mar 20;7:e30134. doi: 10.7554/eLife.30134 (PMC5896883; doi:10.7554/eLife.30134)
Supplement: Supplementary file 1. [file elife-30134-supp1.docx]

| **Parameter** | **Definition** | **Measurement** |
| --- | --- | --- |
| ***λ*** | Mean number of HIV infection attempts per cell. | Total number HIV copies (integrated or unintegrated)/total number cells. |
| ***L_λ_*** | Probability of a cell to live given transmission with *λ* copies at 2 (cell line) or 4 (lymph node cells) days post infection. | Concentration of live cells with λ transmissions/concentration of live cells with no transmission. Concentration of live cells with no transmission is derived in a separate cell culture infected with the identical amount of cell-free HIV but where the infection is inhibited with a high EFV concentration. |
| ***P_λ_*** | Probability of a cell to be infected and live given transmission with *λ* copies at 2 (cell line) or 4 (lymph node cells) days post-infection. | Fraction of live cells positive for HIV dependent GFP expression 2 days post-infection (cell line) or HIV Gag protein 4 days post-infection (lymph node cells). |
| ***q^1^*** | Probability of cell death at *λ*=1. | *-ln(L_λ_)/λ* |
| ***r^1^*** | Probability of cell infection at *λ*=1. | *-ln(1-P_λ_) /λ* |

S Table 1: Parameters and definitions

^1^ Measured in the cell line two days post-infection with wild type cell-free HIV, otherwise fitted using Equation (2).
