# Supplementary material for: Incomplete inhibition of HIV infection results in more HIV infected lymph node cells by reducing cell death
Source: eLife. 2018 Mar 20;7:e30134. doi: 10.7554/eLife.30134 (PMC5896883; doi:10.7554/eLife.30134)
Supplement: Supplementary file 2. [file elife-30134-supp2.docx]

| **Parameter** | **Cells** | **Wells** | **Bands** | **Raw bands/cell** | **Corr. bands/cell^1^** | **P_λ_ or L_λ_** | **Value** |
| --- | --- | --- | --- | --- | --- | --- | --- |
| *r* | 42 | 168 | 24 | 0.56 | 2.4 | 0.49±0.02 | 0.28 ±0.08 |
| *q* | 48 | 192 | 19 | 0.40 | 1.7 | 0.79±0.02 | 0.15±0.07 |

S Table 2: Measurement of *r* and *q*

^1^ Corrected for sensitivity of detection as quantified in the ACH-2 cell-line.
