# Supplementary material for: Incomplete inhibition of HIV infection results in more HIV infected lymph node cells by reducing cell death
Source: eLife. 2018 Mar 20;7:e30134. doi: 10.7554/eLife.30134 (PMC5896883; doi:10.7554/eLife.30134)
Supplement: Supplementary file 3. [file elife-30134-supp3.docx]

| **Participant ID** | **Sex** | **Age**  **(years)** | **LN location** |
| --- | --- | --- | --- |
| 205 | F | 27 | Supraclavicular |
| 251 | M | 32 | Mediastinal |
| 257 | F | 46 | Peribronchial |
| 274 | M | 22 | Peribronchial |

S Table 3: Participant information
