# Supplementary material for: Incomplete inhibition of HIV infection results in more HIV infected lymph node cells by reducing cell death
Source: eLife. 2018 Mar 20;7:e30134. doi: 10.7554/eLife.30134 (PMC5896883; doi:10.7554/eLife.30134)
Supplement: Transparent reporting form [file elife-30134-transrepform.docx]

***eLife’s* transparent reporting form**

We encourage authors to provide detailed information *within their submission* to facilitate the interpretation and replication of experiments. Authors can upload supporting documentation to indicate the use of appropriate reporting guidelines for health-related research (see [EQUATOR Network](http://www.equator-network.org/%20)), life science research (see the [BioSharing Information Resource](https://biosharing.org/)), or the [ARRIVE guidelines](http://www.plosbiology.org/article/info:doi/10.1371/journal.pbio.1000412) for reporting work involving animal research. Where applicable, authors should refer to any relevant reporting standards documents in this form.

If you have any questions, please consult our Journal Policies and/or contact us: [editorial@elifesciences.org](mailto:editorial@elifesciences.org).

**Sample-size estimation**

- You should state whether an appropriate sample size was computed when the study was being designed
- You should state the statistical method of sample size computation and any required assumptions
- If no explicit power analysis was used, you should describe how you decided what sample (replicate) size (number) to use

Please outline where this information can be found within the submission (e.g., sections or figure legends), or explain why this information doesn’t apply to your submission:

Sample size estimation was not determined since this work involves in vitro infection of cellular material, not inferences about a population.

**Replicates**

- You should report how often each experiment was performed
- You should include a definition of biological versus technical replication
- The data obtained should be provided and sufficient information should be provided to indicate the number of independent biological and/or technical replicates
- If you encountered any outliers, you should describe how these were handled
- Criteria for exclusion/inclusion of data should be clearly stated
- High-throughput sequence data should be uploaded before submission, with a private link for reviewers provided (these are available from both GEO and ArrayExpress)

Please outline where this information can be found within the submission (e.g., sections or figure legends), or explain why this information doesn’t apply to your submission:

Below we organize the experiments by the figures they are presented in and detail how experiments were repeated:

Figure 2A-B: To determine the number of integration in our cell line, we performed 3 independent experiments. For the first experiment, 4 cells were used. For the second experiment, 20 cells were used. For the third experiment, 10 cells were used. The combined total was 34 cells. This is stated in the legend of Figure 2.

Figure 2C-D: To determine the number of live infected cells as a function of drug, we performed 3 independent experiments. Each data point from each experiment is based on a minimum of 50,000 cells collected by flow cytometry. Data is presented as means and standard errors of independent experiments. This is stated in the legend of Figure 2.

Figure 3A-B: To determine the number of live infected cells as a function of drug with the L100I mutant virus, we performed 3 independent experiments. Each data point from each experiment is based on a minimum of 50,000 cells collected by flow cytometry. Data is presented as means and standard errors of independent experiments. This is stated in the legend of Figure 3.

Figure 4: To determine the number of live infected cells as a function of b12 with the wild type and L100I mutant virus, we performed 3 independent experiments. Each data point from each experiment is based on a minimum of 50,000 cells collected by flow cytometry. Data is presented as means and standard errors of independent experiments. This is stated in the legend of Figure 4.

Figure 5A: Lymph nodes from 3 different study participants were used to obtain the data. The number of biological replicates possible per lymph node depends on the number of cells in the lymph node. Therefore, we were able to perform only a single replicate of lymph nodes with participant identification numbers (PID) 205 and 257, and three replicates of the last lymph node, PID 251. This is stated in the figure legend of Figure 5.

Figure 5B: To determine the number of integration in lymph nodes, we performed 3 independent experiments. A total of 56 lymph node cells were tested for each drug condition. For the first experiment, 8 cells were used. For the second experiment, 24 cells were used. For the third experiment, 24 cells were used. The combined total was 34 cells. This is stated in the legend of Figure 2.

Figure 6: We had sufficient lymph node material to perform two independent experiments for PID 251. In order to repeat the experiment 3 times for coculture infection, we used lymph node PID 274, of which we had sufficient material for one experiment. We combined the results for both lymph nodes for coculture infection and show the results as Figure 6A for cell-free infection and Figure 6B for coculture infection. This is stated in the figure legend of Figure 6.

Figure 2 – figure supplement 1A-C: To determine the number of integration in ACH-2 cells, we performed 3 independent experiments. A total of 72 cells were tested, 24 cells per experiment. For the third experiment, 24 cells were used. For RevCEM cells, one experiment was performed, and first 12 out of 17 cells assayed are shown. This is stated in the legend of Figure 2 – figure supplement 1.

**Statistical reporting**

- Statistical analysis methods should be described and justified
- Raw data should be presented in figures whenever informative to do so (typically when N per group is less than 10)
- For each experiment, you should identify the statistical tests used, exact values of N, definitions of center, methods of multiple test correction, and dispersion and precision measures (e.g., mean, median, SD, SEM, confidence intervals; and, for the major substantive results, a measure of effect size (e.g., Pearson's r, Cohen's d)
- Report exact p-values wherever possible alongside the summary statistics and 95% confidence intervals. These should be reported for all key questions and not only when the p-value is less than 0.05.

Please outline where this information can be found within the submission (e.g., sections or figure legends), or explain why this information doesn’t apply to your submission:

In Figures 2-6, multiple independent experiments were combined and the data is presented as mean and standard error. For Figure 5C, a 2 tailed t-test was performed to compare DNA copies per cell in the drug and no drug conditions. This is stated in the legend of Figure 5C, and the Matlab script used in the estimation of DNA copies after correction for assay sensitivity is provided as a supplementary data file.

(For large datasets, or papers with a very large number of statistical tests, you may upload a single table file with tests, Ns, etc., with reference to sections in the manuscript.)

**Group allocation**

- Indicate how samples were allocated into experimental groups (in the case of clinical studies, please specify allocation to treatment method); if randomization was used, please also state if restricted randomization was applied
- Indicate if masking was used during group allocation, data collection and/or data analysis

Please outline where this information can be found within the submission (e.g., sections or figure legends), or explain why this information doesn’t apply to your submission:

Does not apply – this study uses human clinical material for in vitro infection purposes only.

**Additional data files (“source data”)**

- We encourage you to upload relevant additional data files, such as numerical data that are represented as a graph in a figure, or as a summary table
- Where provided, these should be in the most useful format, and they can be uploaded as “Source data” files linked to a main figure or table
- Include model definition files including the full list of parameters used
- Include code used for data analysis (e.g., R, MatLab)
- Avoid stating that data files are “available upon request”

Please indicate the figures or tables for which source data files have been provided:

Below are the figures for which source code is provided and the name of the corresponding Matlab file (.m):

Figure 1	script1r2.m

HIV copies (used Figure 2D &3B)	script2r2.m

Figure 2C-D	script3r2.m

Figure 3A-B	script6r2.m

Figure 5C	script7.m

Figure 6A-B	script8r2.m
